# Amplifying voices: The transformative power of voice and voice economy

**DOI:** 10.4102/ajod.v14i0.1742

**Published:** 2025-10-31

**Authors:** Erna van der Westhuizen, Surona Visagie

**Affiliations:** 1 Department of Global Health, Faculty of Medicine and Health Sciences, Stellenbosch University, Stellenbosch, South Africa

**Keywords:** voice, voice economy, empowerment, parents of children with disabilities, participation, representation

## Abstract

**Background:**

Parents of children with disabilities often experience marginalisation and exclusion from decision-making processes. The concept of ‘voice’ as identity and agency is central to addressing these injustices. The Parent Network (PN) and Let’s Talk Parent Tool (LTP) were created to amplify these voices and promote inclusive participation.

**Objectives:**

This study explores the conceptual foundations and practical implementation of the PN and LTP. It investigates how these tools empower parents, particularly mothers, to engage in policy dialogue and advocate systemic change.

**Method:**

Using a qualitative descriptive approach, data were gathered in July–August 2023 from two purposively selected key informants and supporting grey literature. A WhatsApp-based focus group enabled rich, reflective responses. Data were thematically analysed using ATLAS.ti, with iterative coding to identify themes.

**Results:**

Themes revealed the PN’s role in reducing isolation, enhancing self-representation and building parent-led networks across all South African provinces. The LTP tool supports policy monitoring by allowing users to document accessibility barriers. Offline functionality and multilingual support increase accessibility for digitally marginalised families.

**Conclusion:**

The PN and LTP create transformative spaces where parents reclaim their voices and influence decisions that affect their lives. These tools address systemic inequities by promoting emotional safety, digital inclusion and local leadership.

**Contribution:**

This study contributes a replicable model for digital voice empowerment. It highlights how community-driven platforms can support participatory policy development and build a ‘voice economy’ that values lived experience as a catalyst for social transformation.

## Introduction

Empowerment enables individuals to become agents of their own progress, fostering equality and participation within inclusive societies. Central to this process is the concept of ‘voice’, which reflects identity, agency and self-representation. Marginalised groups, particularly caregivers of children with disabilities, often experience exclusion from decision-making processes. Reclaiming their voice and agency empowers them to advocate for their rights and influence policy decisions for systemic change. The ‘voice economy’ further emphasises the collective value generated when individuals share their experiences and perspectives, contributing to social transformation (Van der Westhuizen & Visage 2025). However, these caregivers are often excluded from formal decision-making processes and lack platforms through which their lived experiences can shape public discourse or policy. In response, tools like the Parent Network (PN) and Let’s Talk Parents (LTP), developed by the Shonaquip Social Enterprise (SSE),^1^ promote this by enabling parents to engage in decision-making and policy monitoring, bridging gaps in implementation and fostering sustainable impact. This article explores the conceptual basis and operational implementation of the PN and LTP.

### Participation and representation: Voice matters

Interaction is essential for participation, and voice, in its universal nature, is a key facilitator of this interaction. It offers a personalised medium through which individuals can engage, share ideas and come to agreements (Bhatti et al. 2020). In democratic nations like South Africa, this voice is not only symbolic but institutionalised. The Independent Electoral Commission (IEC), for example, encourages citizens to vote, positioning their voice as a mechanism for decision-making that shapes one’s life and country. Similarly, policy processes such as White Paper on the Rights of Persons with Disabilities WPRPD (Department of Social Development [DSD] 2016) invite citizens to use their voices to influence governance.

Using your voice implies ‘owning your voice’. This concept involves expressing yourself, forming and sharing opinions, vocalising your thoughts and being genuinely heard and seen. In alignment with the human rights approach to disability, it can be argued that using one’s voice is a critical aspect of exercising and claiming rights (Masferrer 2023; Thambinathan & Kinsella 2021). Being part of a community where one feels confident in self-expression signifies a sense of belonging to that community, both essential for fostering representation and voice (Cobigo, Martin & Mcheimech 2016).

However, owning a voice is not guaranteed for everyone. Marginalised communities often lack sufficient self-representation, receiving minimal attention from both their own communities and prominent figures. They are frequently subjected to bias and discrimination, even when their societal contributions are noteworthy. Misrepresentation occurs when the standards, values and voices of those in authoritative positions are taken as the norm. These circumstances hinder the potential for improving the active participation of marginalised groups, such as people with disabilities (Chowdhury 2022; Leng, Huang & Zhou 2024).

#### Approaches to disability and voice empowerment

The evolution of various disability approaches has influenced voice and representation, shaping marginalised groups’ perception of their right to assert agency. The medical model promotes curative interventions and impairment-focused rehabilitation to achieve a perceived level of ‘normality’. In the process, the medical model reinforces patriarchal authority and grants excessive control to so-called expert professionals over the lives of people with disabilities (Hussey et al. 2017), silencing and disowning voices (Carling-Jenkins 2016; Ned 2022).

On the contrary, the human rights approach emphasises dignity, equal opportunity and non-discrimination, advocating for full participation in society (Ngcobo & Gumede 2022). The human rights framework emphasises the value of inclusive participation in overcoming historical power imbalances, shifting from privileging the voices of a select few to embracing the diverse perspectives of all.

Author and storyteller Chimamanda Ngozi discusses how the concept of a ‘single story’ shapes our self-presentation and our impact on others. She uses the term ‘single story’ to describe the inaccurate perceptions and oversimplified understandings of individuals, groups or communities resulting from a singular narrative:

‘The single story creates stereotypes, and the problem with stereotypes is not that they are untrue, but that they are incomplete … they make the one story become the only story.’ (TED 2010: 13:13/19:16)

Voice empowerment embraces the narratives of marginalised voices and facilitates the shift from a single story to a more comprehensive narrative. This not only reduces social exclusion but also opens the door to sharing constructive ideas aimed at enhancing the rights of people with disabilities and their families.

It is, therefore, crucial to consistently listen to and learn from those most affected by social issues. Voice empowerment involves validating their lived experiences and empowering them to lead the driving change. It is essential to believe in their stories, provide platforms for their voices and share their experiences without making ourselves the focal point. This includes examining who gets represented, who can voice their opinions, and how and by whom they are represented in the policy formation process.

Developing the confidence and voice to transform daily realities requires a multifaceted approach that encompasses systemic changes, inclusive empowerment and the creation of an environment rooted in inclusivity and equity, where marginalised voices are actively sought out, heard and respected. Rather than imposing our opinions, we should ask about the resources, community assets and systems that genuinely support marginalised groups such as persons with disabilities (Hutcheon & Lashewicz 2019; Ned 2022). Therefore, voice empowerment enables individuals to assert their rights and shape the decisions, policies and conditions that impact their lives. A human rights-centred approach acknowledges everyone as agents of their own progress, advocating for equality, participation and inclusion (Borry & Reuter 2022). This approach not only promotes moral integrity but also accelerates progress toward equitable and sustainable development. It highlights the transformative potential of human rights in driving development agendas. Additionally, accountability and remedial actions for human rights violations are crucial, necessitating robust laws, policies, institutions and administrative procedures to address and rectify injustices (Brear 2020; Browne & Millar 2018).

#### Voice economy

Empowered voices emerge from environments where individuals feel emotionally safe and included, fostering a sense of agency, self-determination, and representation. This creates a system where the expression and exchange of individual and collective voices generate collective value, drive social transformation, and address systemic barriers like ableism. A voice economy emphasises strengths-based practices, inclusivity, collaboration, and the use of digital technologies to ensure all voices are heard and valued, fostering equity and social justice (Van der Westhuizen & Visagie 2025).

Similar to how conventional economies create value by efficiently allocating resources to produce goods and services, social collective value can be created when marginalised individuals, such as parents of children with disabilities, are given platforms to express their experiences, opinions and knowledge. This results in innovative solutions and social transformation. A voice economy, therefore, emphasises strength-based practices, inclusiveness and collaboration, viewing contributions as valuable assets that can influence policy, build social capital and foster self-efficacy, ultimately leading to meaningful and impactful outcomes (Van der Westhuizen & Visagie 2025). Voice economies play a crucial role in challenging power hierarchies by recognising and validating contributions from all individuals, particularly marginalised groups (Van der Westhuizen & Visagie 2025).

Collective voices have the power to influence critical aspects of people’s lives, fostering personal transformation by enabling them to express themselves and assert their positions within societal structures. This provides the opportunity to challenge power imbalances, reshape societal identities and roles and counteract dominant narratives through local and national advocacy.

#### Virtual communities, smart applications and voice

Digital technologies, including apps like WhatsApp, are emerging as strategies for marginalised groups to amplify their voices (Magee, Leman & Balasubramaniam 2024; Ortiz et al. 2019). Lievrouw (2011:2) describes digital technologies as ‘inexpensive, powerful tools’ that provide a platform for expressing viewpoints that might otherwise remain unheard or suppressed in public discourse. However, connectivity and accessibility to such technologies are uneven and shaped by socio-economic factors, with individuals from low-income or rural communities often facing barriers to data, devices and reliable internet access (Colom 2020; Gogginf & Ellis 2020). Consequently, these technologies facilitate social movements, empower previously excluded individuals to voice their concerns, coordinate campaigns for change, mobilise the public, document injustices and disseminate information to broader audiences (Anderson et al. 2021; Herawati, Maro & Widowati 2024; Ortiz et al. 2019).

The potential of digital applications to foster participation and amplify voices offers reassurance to parents of children with disabilities (CWD) and other marginalised groups that their voices are important and can bring about change. By utilising technology to elevate these voices – especially when framing inequalities from a grassroots perspective – it becomes evident that the effectiveness of national and local agencies is often limited without the support and commitment of community and voluntary groups (Welch et al. 2023). Moreover, digital platforms allow individuals unable to attend physical meetings, such as parents of CWD, to have their voices heard, thereby enabling broader participation and amplifying a greater diversity of voices (Olanrewaju et al. 2021; Van Toorn & Cox 2024). This, in turn, fosters a greater sense of ownership within the process.

#### Mothers (families) of children with disabilities and voice

Caring for CWD usually falls on the mother or other women in the family (Vadivelan et al. 2020). For this article, however, we embraced South Africa’s diversity of family forms and went beyond ties of blood, marriage, kinship and legal arrangements. We use the terms ‘family’ and ‘mothers’ in the context of social connections and identity ties, which extend beyond a particular physical residence (DSD 2013).

Mothers and fathers of CWD experience more psychological and physical challenges than their peers without CWD, including sleep deprivation, headaches, fatigue and muscular pain, to name a few (Chen et al. 2023; McLorie, Hackett & Fraser 2023; Woodgate et al. 2024). They face isolation because of social stigma and prejudice (Mkabile & Swartz 2020; Ngubane-Mokiwa 2018; Walters, Britz & Van der Westhuizen 2020), which often disempowers them. They navigate life with limited knowledge and support. Their efforts to access information and services are typically driven by individual responses to disenfranchisement rather than a facilitated process supported by policy implementation (Holness 2022; Molokwane & Tshombe 2018; Ngcobo & Mabuyi 2022; The Presidency 2013).

Parents frequently find themselves marginalised and voiceless when advocating for their child’s well-being, being dismissed or overlooked by institutions, educators and healthcare providers (Walters et al. 2020). This not only deprives parents of their rightful role as advocates but also removes the opportunity for the broader community to benefit from crucial perspectives that could lead to more inclusive and effective support systems for CWD (Antwi 2023; Cowhy, Mulroy & Bonilla 2024; Rossetti et al. 2020).

The prevalence of the medical model of disability (World Health Organization [WHO] 2001) exacerbates their experiences of exclusion and voicelessness (Hussey et al. 2017). The prioritisation of professionals who are more educated, respected and influential in decision-making has resulted in professional gatekeeping to resources (Carling-Jenkins 2016; DSD 2016), enforcing class systems and unequal power relations in society (Ned 2022).

#### Problem

Parents’ voices are often overlooked in society and policy development, despite their intimate familiarity with their children’s unique requirements through lived insights and invaluable experiences (Guo et al. 2014). Instead of being advocates for their children and drivers of their futures, they are left without a voice and choice (Walters et al. 2020). Even so, ironically, in numerous countries, it is through the lobbying efforts of parent associations that essential services have started to be extended to families of CWD (McDiarmid, Pineda & Scothern 2021). Through this active engagement, parents play a central role in influencing policies that directly affect their lives and communities (Holness 2022; Vivier & Sanchez Betancourt 2023). In South Africa, the experience of being voiceless and disempowered led to a digital PN and LTP tool. The PN and LTP are pioneering strategies in South Africa, and possibly Africa. While anecdotal evidence and stakeholder interest highlight their value, and 2 years of operation and growth suggest sustainability, neither has yet undergone formal evaluation. A PhD study evolved to describe the digital PN and LTP tool, user experiences and satisfaction with it, and to explore if these applications could provide a framework for voice and voice empowerment. This article describes the conceptual framework and processes of the PN and LTP. Future work explores user satisfaction with the PN and LTP and the role the PN and LTP play in the lives of mothers of CWD.

#### The parent network and let’s talk parents tool

In response to the need for support and connection among families of children with disabilities, SSE (2021) launched a PN (SSE Annual Report 2022 and 2023). This digital network leverages existing technology to enable parents of CWD to connect and address their challenges across all nine provinces of South Africa. By utilising smartphones, parents participate in training and support sessions via WhatsApp. The PN’s work is rooted in participatory practices, empowering parents to actively influence decisions that affect their lives and to help identify solutions to the barriers they encounter. The model is specifically designed to address the marginalisation of voices by promoting meaningful participation. As parents’ awareness of disability policy increased through the PN, their need to understand and engage in monitoring policy became evident. To address this need, a monitoring tool, the LTP tool, was created as a sub-section of the PN to facilitate the user-friendly monitoring of the implementation of the South African WPRPD as part of the PN (SSE Annual Report 2022 and 2023).

## Research methods and design

This study formed part of the first author’s PhD. A transformative paradigm guided the research, as it emphasised advancing social justice and addressing power inequalities (Mertens 2012). Central to this approach are ongoing dialogue, power-sharing, mutual respect and collective action, which were essential components of the study methodology (Godden 2017). A Steering Committee was established to guide the research. It included three representatives from the study population, two from SSE, the first author’s PhD supervisor, and the lead author. The committee ensured the study’s practical relevance helped mitigate bias stemming from the lead author‘s involvement in the PN and LTP initiatives. The PhD supervisor provided academic guidance, including methodological support and oversight of data analysis. The Steering Committee contributed extensively to shaping the study’s questions, aims, objectives and methods. Communication occurred through WhatsApp and Zoom meetings, and all correspondence was saved as part of the study’s audit trail.

This article describes the PN and LTP through the eyes of two purposively selected key informants. The two key informants held comprehensive knowledge and insights into the PN and LTP tools. Key Informant 1 (KI1) provided insights from a dual perspective – both as a parent of a child with disability and as an administrator within the PN. Key Informant 2 (KI2) contributed perspectives from her role as a project manager. She also co-designed the LTP tool alongside the first author. Additional data were gathered from virtual and physical documents related to the LTP and PN. Finally, the reflections of the first author, the co-founder of the PN and LTP, were a source of data.

A qualitative descriptive design was used to gather information to describe the conceptual framework and processes of the PN and LTP (Bradshaw, Atkinson & Doody 2017). After obtaining informed consent, data were collected in July 2023 and August 2023, in English, via a WhatsApp focus group. The WhatsApp group included the two key informants and the first author. All information was treated confidentially. Both participants expressed a preference for responding to questions via WhatsApp rather than participating in semi-structured interviews. This method allowed them more time to reflect on and consider their answers before responding.

The data were analysed using an inductive thematic approach, with the support of ATLAS.ti software. The data were thoroughly reviewed to capture the respondents’ perspectives, and initial codes were assigned and recorded within ATLAS.ti. As the analysis progressed, the initial set of codes was modified, recognising that human interpretation extends beyond the capabilities of software programs (Linneberg & Korsgaard 2019; Locke, Feldman & Golden-Biddle 2023). During the analysis, some themes were merged, while others were newly formulated to ensure they accurately reflected the data (Maguire & Delahunt 2017; Nowell et al. 2017). This careful refinement of themes ensured that the analysis remained true to the participants’ experiences and insights (Maguire & Delahunt 2017; Nowell et al. 2017). Member checking took place.

### Trustworthiness

Several strategies were implemented to ensure the study’s trustworthiness. The researcher maintained a reflective stance throughout because of close involvement with the PN and LTP. Measures included forming a diverse Steering Committee, applying theory-based tools, and appointing an independent research assistant for aspects of data collection. The Steering Committee played a critical role in shaping the study design and ethical protocols. Credibility was further supported through member checking, triangulation with document data and iterative analysis. Dependability was enhanced through transparent documentation and the use of an audit trail. Confirmability was strengthened by reflexive journaling and committee feedback, while transferability was supported through rich descriptions of context and participants.

### Ethical considerations

The study received ethics clearance from the Health Research Ethics Committee at Stellenbosch University on 23 March 2023 (HREC Reference No: S23/01/010 [PhD]). Participation was voluntary, and no data were collected before written informed consent was obtained. Participants could benefit from the study if their insights contribute to improving how the PN and LTP serve communities. Anonymity and confidentiality were maintained throughout.

## Results

In line with the study’s aim of examining the conceptual foundations and practical implementation of the PN and LTP, the thematic analysis identified four overarching themes: (1) the ‘accidental’ start of the PN, (2) the PN as a virtual network addressing parental isolation and fostering capacity building, (3) roles within the PN and (4) the operations of the PN (Table 1).

**TABLE 1:** Emerging themes and sub-themes.

Theme	Sub-theme
1. The ‘accidental’ start of the PN: A reflection	-
2. The Parent Network: A virtual network for parents of children with disabilities to address parental isolation and capacity building	-
3. Roles in the Parent Network	3.1 Advisory committee 3.2 Network Parents 3.3 Parent champions
4. The operations of the Parent Network	4.1 Provincial WhatsApp group 4.2 Interprovincial WhatsApp training groups 4.3 Network Parent WhatsApp group 4.4 Let’s Talk Parents Tool

### Theme 1: The ‘accidental’ start of the parent network: A reflection

In December 2018, while undertaking a fellowship with the Atlantic Institute on a health equity advocacy project in South Africa, the lead author faced challenges in collecting data needed to understand out-of-pocket costs for people with disabilities (PWD) and their families. These challenges included being unknown to participants, a lack of community entry paths, and limited access to parents of CWD because of their isolation. Additionally, parents were often reluctant to participate, perceiving the researcher as an outsider and a ‘professional’, which hindered trust building:

‘Such professionals are perceived as being more educated, engendering respect and having influence with greater control over access to resources. This can reinforce class systems and unequal societal power relations that we need to overcome.’ (Atlantic Fellows 2023:n.p.)

The turning point came when the researcher collaborated with 14 trusted community leaders, including parents of CWD, who were already embedded in their local community and possessed strong facilitation skills. A WhatsApp group was created for collective problem-solving and data collection. This participatory approach empowered community leaders and led to meaningful conversations about disability-related issues: ‘creating an environment where people can develop their capabilities, live together in peace and feel a sense of belonging and ownership’ (Atlantic Fellows 2023:n.p.)

After the project concluded, discussions on the WhatsApp group continued, highlighting the need for ongoing support to parents of CWD. This led to the experiment of launching a virtual network to provide a platform for parents to connect, share experiences and support each other in raising CWD. This marked the beginning of the PN.

The PN’s design embraced a transformative paradigm, shifting away from hierarchical power dynamics and drawing on the strengths and experiences of parents who had first-hand knowledge of the challenges they faced:

‘It is really … how do we reach far more families in South Africa by doing it with parents, in partnership with them and not thinking that we must be the people to do that always.’ (Atlantic Fellows 2021, 03:19)

‘Parents know enough; they got specific skills and experiences.’ (Atlantic Fellows 2021, 01:43)

As word spread about the PN’s existence and value, more parents requested to join the WhatsApp group as a space where their voices mattered: ‘Decades of power have isolated them and silenced their voices. Breaking this isolation is critical’ (Atlantic Fellows 2023:n.p.)

**FIGURE 1:**
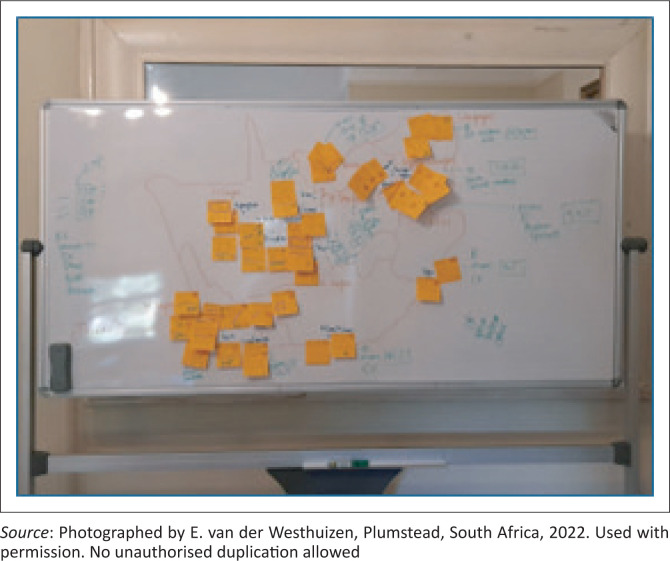
First mapping of the network, small enough to do it manually with sticky notes.

This led to the network’s formal establishment in December 2019, with approximately 100 parents (Figure 1). In the early stages, the network was of such a limited scale that parents could be manually plotted on a map using sticky notes:

‘The parent network is a catalytic community of parents of children with disabilities that are coming together, organising themselves in a particular way to get information from really grassroots level, lived experiences of people. Really it is about creating quality information and then share the information to parents of children with disabilities.’ (Atlantic Fellows 2021, 01:14)

To practice transformative principles for voice empowerment and ensure the effective design and implementation of the network, an advisory committee was formed in 2020. The committee comprised five members: two fieldworkers and parents from the Eastern and Western Cape economic studies and three identified through SSE programmes from the Northern Cape and Gauteng. The intention was to have representation from several provinces and to ensure that diverse voices direct the development of the PN. This committee played a crucial role in guiding the Board of Trustees and facilitating discussions on the network’s model and design, which were rooted in principles of social justice inspired by the work of Sensoy and DiAngelo (2009). These conversations continued to be guided, with ongoing advocacy for the purpose and role of the PN.

As the network continued to grow, the advisory committee naturally dissolved in 2020, reflecting the organic development and empowerment of the community. I acknowledged this as part of the social innovation process and believed that the committee had served its purpose and that we were entering the next iteration of the PN. Network parents emerged, taking on key roles in driving the network’s initiatives and shaping its future. At this point, the network had grown from the initial 14 parents to 122.

### Theme 2: The Parent Network: A virtual network for parents of children with disabilities to address parental isolation and capacity building

The network addresses the critical need for support and connection among families of CWD. By encouraging active participation and empowering parents, it enables them to have a stronger voice in decisions impacting their lives, as highlighted by key informants and myself:

‘It is very important to have such network, as us, most of the parents who are raising children with disability, we don’t have much information about disability.’ (KI1, parent of CWD, administrator, F)

‘Through this [*PN*] they are able to support one another, connect with one another and really address barriers around isolation for parents of children with disabilities.’ (KI2, project manager, OT, F)

‘Through working with groups, chiefly the parents of children with disabilities, I have discovered that building networks of support is crucial to giving confidence to marginalised groups. For instance, in the case of parents of children with disabilities, decades of power have isolated them and silenced their voices.’ (Atlantic Fellows 2023:n.p.)

The PN operates as an e-community of practice, ‘a remote network of parents with children with disabilities, as well as persons with disabilities … connected to one another … over the platform of WhatsApp’ (KI2) (cf. SSE 2020/2021 & 2021/ 2022).^2^ ‘We have also setup a mobile education and social connection platform for parents of kids with disabilities which we call the parent network’ (Atlantic Fellows 2021, 01:07). The choice to use a virtual platform was driven by a wish to be inclusive, ‘The Network operates remotely as we have people or parents across South Africa as we don’t want to leave anyone behind’ (KI1, parent of CWD, administrator, F).

**FIGURE 2:**
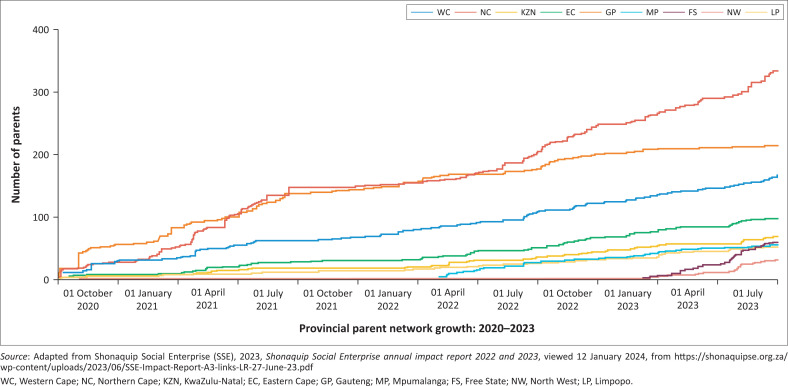
Parent network growth per province.

As noted by KI2 and supported by the SSE Annual Impact Report (2022 and 2023), the PN operates across all nine provinces of South Africa. The network expanded from 14 parents in early 2019 to over 1200 by September 2023. Figure 2 illustrates that as of September 2023, the Western Cape group comprised 203 parents, Free State 58, Northern Cape 259, Eastern Cape 110, KwaZulu-Natal 73, Gauteng 394, Mpumalanga 57 and Limpopo 52.

### Theme 3: Roles in the parent network

Key Informant 2 emphasised the importance of establishing a clear structure and defining specific roles within the PN to effectively operationalise the network:

‘We kind of were quite aware in the beginning that in order for this community care approach to work and in order to move towards the network being more sustainable over time we would really need leadership to develop from within the network. Specific roles that needed to happen in terms of how information flows in the network, so how information comes from families and how it goes back into the network and back to families and so to do that, we needed a group of leaders of parents in the network and those have been our network parents.’ (KI2, project manager, OT, F)

**FIGURE 3:**
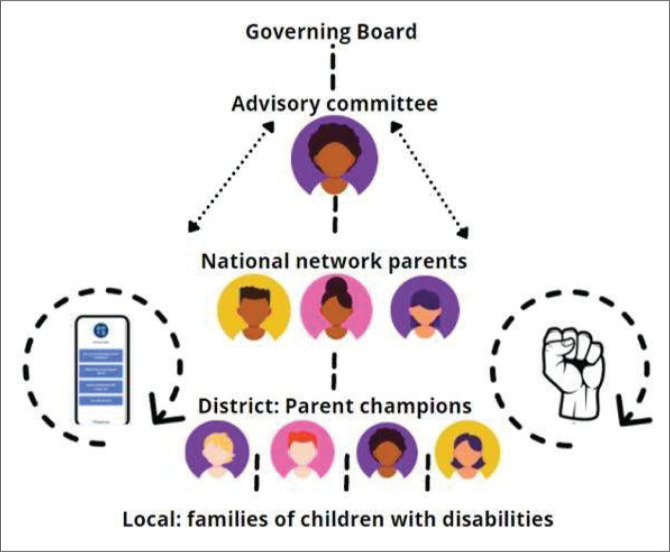
The conceptual model of the parent network.

The specific roles included an advisory committee, network parents (the group of leaders mentioned by KI2), parent champions and advocacy partners (Figure 3). These roles were intentionally designed to leverage parents’ strengths and implement the community care approach (SSE Annual Impact Report 2020 and 2021).

#### Sub-theme 1: Advisory committee

At the inception of the PN, an advisory committee was established to co-design and later pilot the LTP tool. The committee ensured that parents’ voices were represented at the SSE board level, thereby influencing decision-making processes. In 2020, the advisory committee dissolved as the network of parents became well-established. From that point onward, the network parents took on the responsibility of driving initiatives and shaping the network’s future (SSE 2019 and 2020).

#### Sub-theme 2: Network parents

The network parents (either parents or caregivers of CWD) are at the heart of the PN. They act as leaders within the network. Their roles have evolved organically over time in response to emerging needs, as described by Key Informant 2:

‘It has developed over time quite naturally and I think that that’s really something that makes the network quite special. It has had very organic growth and as the needs have changed and adapted or developed, we’ve been able to adapt the roles and we’ve been able to adapt the method in the way which the network works to meet the needs of the individual and of the whole.’ (KI2, project manager, OT, F)

Network parents facilitate the flow of information within the network. They play an instrumental role in gathering information from families without smartphones or good connectivity through door-to-door visits and relay it to WhatsApp groups:

‘Network parents conduct door-to-door visits which are a key enabler to connect with other parents, complete the LTP tool, share information about the network and therefore be the catalysts in the flow of information.’ (KI2, project manager, OT, F)

Network parents play a vital role in providing parents with essential information and ‘know-how’ to make informed decisions regarding the care and future of their children. They facilitate referrals when necessary. Their ability to fulfil this role was developed through training provided by the SSE team and the broader network, which will be discussed under the section ‘Training All WhatsApp Groups’. Key Informant 2 explained:

‘So for instance if we do a session around say feeding in the network and that network parent visits a family and sees that the child is maybe not positioned correctly and they are aspirating while they are eating, that network parent will have the knowledge to say you know, it looks like your child is not seated safely and not swallowing and they are not eating safely. I think we need to do a referral to a speech therapist at the local clinic, or here is a different type of way that you can position your child that might be better to feed them in the meantime … [*PN*] make referrals to social, educational, and health services, and use the LTP tool to monitor the implementation of policies, also on behalf of those without phones or connectivity.’ (KI2, project manager, OT, F)

Network parents guide decisions based on the needs they identify within their provincial groups and through their observations in the field. For instance, they might notice that parents are unaware of their ability to access hospital transport. This issue is then discussed in their dedicated WhatsApp group, after which the relevant information is shared across the provincial groups and the ‘Training All’ WhatsApp groups. Their role in identifying needs and facilitating responses is crucial to the PN’s ability to respond appropriately.

Recently, network parents have taken the initiative to lead conversations within their provincial WhatsApp groups. As Key Informant 2 explained:

‘network parents have volunteered additionally as provincial WhatsApp volunteers, and their role is to support the provincial WhatsApp groups and kind of get the conversation going among the provincial groups.’ (KI2, project manager, OT, F)

By being visible in the field, network parents also play a pivotal role in driving the growth of the network:

‘Through their work, they raise awareness of the parent network … Network parent meets another parent at the local clinic, at the SASSA offices, in the mall and tells them there is this network for parents with children with disabilities, you can join it … it is also really a big part of what the network parents do in doing door to doors and kind of identifying new families and then referring them to the network.’ (KI2, project manager, OT, F)

Including parents who are either not part of the PN or lack access to smartphones remains a priority, as the SSE team is particularly aware of the connectivity challenges in rural South Africa. Network parents play a crucial role in bridging this gap by facilitating the flow of information to these families. They ensure that information extends beyond the formal network structure. As Key Informant 2 explained, ‘families who aren’t able to access the network through these groups benefit by doing door-to-door family support visits’ (KI2, project manager, OT, F). Key Informant 1 added:

‘Different roles of our Network groups, e.g., on the NP group we get all the information of people and children we can’t reach as maybe those families are way too behind the technology model as this group goes to far as villages to try their best to bring them services that they can’t reach them.’ (KI1, parent of CWD, administrator, F)

**FIGURE 4:**
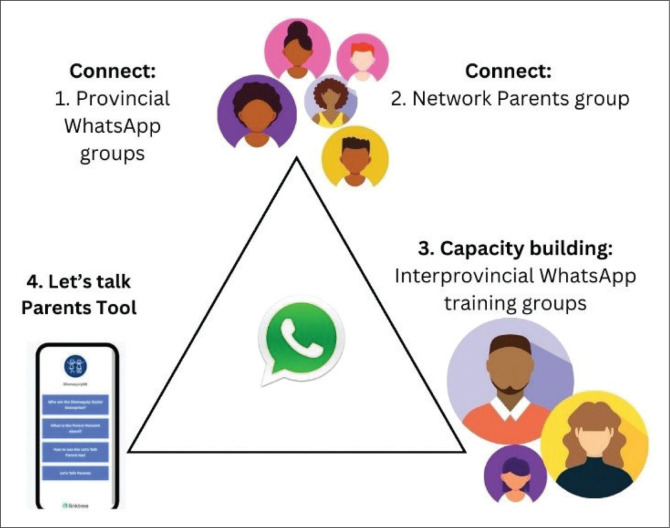
The four components of the PN: WhatsApp groups and the Let’s Talk Parent tool.

Network parents are compensated for their time and receive a travel allowance to cover the costs of using their preferred mode of transport when visiting families within their geographical areas. They are also provided with mobile data to contact families for appointments, seek advice from the SSE team or discuss any questions. Additionally, they are supported with workshop materials, PN posters, SSE t-shirts and identity cards (Figure 4). As Key Informant 2 explained:

‘We were only really able to start providing 18 network parents with a volunteer stipend last year [*2022*]. Before that, all the work that the network parents did, who are kind of the leaders in the network, was all on a volunteer basis.’ (KI2, project manager, OT, F)

A parent who is a member of the PN can apply to become a network parent. Applicants commit to conducting door-to-door visits within their districts, using the LTP tool with families, conducting research under guidance, remaining active in the provincial and ‘Training All’ groups, and facilitating LTP community dialogues in their communities (SSE Annual Impact Report 2022 and 2023; Van der Westhuizen & Visagie 2025). Currently, 70 network parents operate across 31 districts in nine provinces.

#### Sub-theme 3: Parent champions

Parent champions are parents who advocate for their own children and others. Typically, most families in the PN play this role. Network parents connect with parent champions and coordinate the information they provide. As parent champions gain confidence, they start identifying the topics they want to discuss or the training and support they need (SSE Annual Impact Report 2020 and 2021).

### Theme 4: The operations of the parent network

The essence of the PN comprises four interconnected components (Figure 4). These are:

- Provincial WhatsApp chat groups where parents from the same geographical area connect.
- Interprovincial WhatsApp training groups for capacity building and sharing of information by professionals and others in the know.
- Network parent WhatsApp group.
- The LTP tool for monitoring policy implementation.

The first three different WhatsApp groups each address a particular need, as explained by Key Informant 1:

‘We have 3 different types of groups in the Network, which are Training All group: where all of our sessions [*are*] being held … the group that accommodate everyone. Provincial Groups: is where we add our parents in their different province so that they can know each other and build some friendship for themselves so that in future they will maybe go out on play dates with their children and plan their own awareness. The Network Parents group: this is one for the 70 parents who do the volunteering work for the Network, those who do our research and door to door parties and obviously help the Network to grow.’ (KI1, parent of CWD, administrator, F)

#### Sub-theme 1: Provincial WhatsApp groups

**FIGURE 5:**
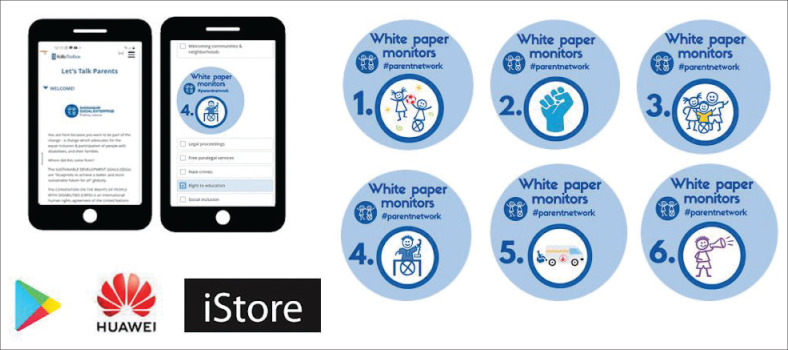
Visual of the let’s talk parent and pillars available to monitor.

The nine Provincial WhatsApp chat groups form the foundation of PN operations and serve as the initial point of contact for any parent joining the network (Figure 5). WhatsApp was chosen because of its widespread familiarity and ability to facilitate connections across time and place, while overcoming the limitations and expenses associated with physical travel. These groups enable parents to connect despite transport and financial barriers, as well as the challenges of caring for a child with disability, which often requires 24 hours a day, 7 days a week attention. WhatsApp allows network parents to share updates with the entire PN and easily organise smaller discussion groups on topics important to them (SSE Annual Impact Report 2022 and 2023):

‘Provincial group is where all the daily activities happen as we have all different things going on like they shared birthdays, awareness, love, cries and grief. That is where we feel the importance of this Network as we get all the type of support we need there.’ (KI1, parent of CWD, administrator, F)

As articulated by Key Informant 2, the Provincial WhatsApp chat groups are entirely parent-driven:

‘They are kind of led by parents themselves, so they are allowed to lead the conversation in any way they feel is beneficial to them and are a way for parents to access support, to access services, to access referrals amongst one another but also from support of the team.’ (KI2, project manager, OT, F)

Being parent-led, Key Informant 2 adds that the PN has:

‘dedicated provincial WhatsApp volunteers who are parents themselves, who support those provincial groups with translations, because language can often be a complex element to consider as, just because parents are from the same province, does not mean they speak the same language.’ (KI2, project manager, OT, F)

The groups have a particular onboarding procedure, in accordance with the *POPIA Act*, as described by Key Informant 1:

‘So there is that whole process and then the parent gets added to both the provincial group and to the training all groups so they can be a part of that two aspects of the network. Parents can also directly message [PN administrator] and request to be added. We have group rules which are shared in the groups so that parents kind of are orientated in terms of what are the rules and what they can and what they cannot expect in these groups. And then we have welcome orientation packs for the parents so when they are added they receive more information about what the parent network is. There are sessions we run more frequently in the network on how to use the Let’s Talk Parents tool.’ (KI1, parent of CWD, administrator, F)

#### Sub-theme 2: Interprovincial WhatsApp training groups

Interprovincial WhatsApp training groups operate as ‘training rooms’, which are opened twice monthly to offer training sessions. The Training All WhatsApp groups function across provinces, and financial support to attend is sometimes provided, depending on the availability of funds. Key Informant 2 explained:

‘We have parents from all provinces connected to one another and those we use bi-monthly for training sessions and those remain closed [*outside of training sessions*] to encourage conversation and engagement to happen on the provincial groups … dependent on funding through investors … there are periods when we are able to provide data for parents to training sessions.’ (KI2, project manager, OT, F)

The training sessions focus on building capacity in various disability-related topics, including different types of disabilities, the social model of disability, advocacy skills and guidance on relevant legal and policy frameworks. Facilitators are invited by the SSE team and selected for their topic-specific expertise from both public and private organisations. Key Informant 1 (KI1) commented the following:

‘There’s session that happens in the group that helps us a lot to know more about different types of disabilities and we have guests that are professional who lead those sessions … we get all the most important/advice from our professionals that runs the session for our Parents, the information that help us to know the way forward also what you can do while waiting at home for the medical assistance if your child/person is maybe having seizures we get all that kind of information from that group.’ (KI1, parent of CWD, administrator, F)

The identification of training topics is driven by parents. Conversations within the provincial groups and insights from network parents are closely monitored to track recurring questions and referrals. This approach helps identify topics that could have broader relevance and provide value to the entire PN, as described by Key Informant 2:

‘If in a provincial group we notice that a lot of parents are requesting information around how to position their child appropriately in a wheelchair or if they don’t have a device like an appropriate wheelchair, how can they position their child better for feeding for instance, then we will invite maybe a seating practitioner to come do a session around positioning, we might invite a speech therapist to do a session around feeding, and so we use those platforms for really kind of for educational purposes, for sharing knowledge, for skills building and then there are very strong elements of advocacy as well.’ (KI2, project manager, OT, F)

The Training All sessions became a cornerstone of the work of the PN and are widely shared with parents, even beyond the network.

#### Sub-theme 3: Network parent WhatsApp group

The network parents have a dedicated group where they, as the leadership of the PN, connect to share information, ask advice from each other and support each other. The group provides a platform for discussions on practice, advocacy and the general operations of their work. The SSE team uses this group to gather input from network parents on PN-related decisions and to obtain feedback from funding partners.

#### Sub-theme 4: Let’s talk Parents tool

The LTP tool is the fourth component of PN operations (Figure 5). It monitors the implementation of the WPRPD (DSD 2016). The tool empowers parents to identify and select issues under each strategic pillar of the White Paper where they believe policy implementation is lacking, serving as both an advocacy and policy monitoring tool (SSE Annual Report 2022 and 2023). As Key Informant 1 explained:

‘The LTP I can say it’s the tool that monitor the barriers that we face everywhere we go like you went to the Mall of your choice and you see there’s no parking for people with disability you take out your phone and open your app and record that barrier.’ (KI1, parent of CWD, administrator, F)

Initially, the LTP was designed as a Google Form. During the pilot phase, however, parents reported practical challenges, such as high data requirements. As one key informant explained:

‘The Let’s Talk Parents tool actually started out a lot more simply, we trialled it first, as a Google Form that parents could complete … access to data and network is a serious barrier for families and so having something that is available offline was very important. Already at that stage we were thinking about how we can make this more user-friendly for families.’ (KI2, project manager, OT, F)

**FIGURE 6:**
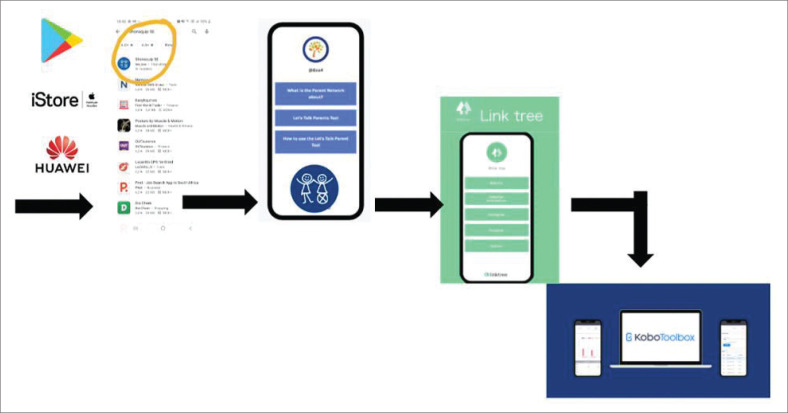
Let’s talk parent technological design.

To address these challenges, various iterations were tested, including a ‘please call me’ service. However, a more sustainable solution was needed. To overcome data limitations and user challenges, the SSE subscribed to Andromo.com, a platform that enables the creation of Android applications without requiring coding or programming expertise. This decision led to the use of Andromo (Figure 6). Because Andromo provides a customisable shell application, a Linktree platform was created to host and present the LTP tool in an app format (Figure 5).

The LTP was eventually set up using Kobo Toolbox, a widely-used open-source platform for data collection, management and visualisation, particularly suited for research and social good (Figure 6). As Key Informant 2 noted, ‘and eventually landed on some funding, getting it using a Kobo tool which works like a link on our SSE app’. Kobo Toolbox was selected because it operates offline, making it ideal for the context in which the LTP is used. As Key Informant 2 further explained, the …:

‘tool is available to use off-line so parents can, for instance if they don’t have access to a connection or network, they are able to submit the LTP off-line … we can confidently say that the parent can be in the middle of nowhere and submit a LTP, it’s not going to get lost and it’s going to come through to us.’ (KI2, project manager, OT, F)

Currently, the LTP tool is accessible through the SSE application, which can be downloaded from the Google Play Store, iStore and Huawei App Gallery. It is available in five languages: English, Afrikaans, isiXhosa, isiZulu and Setswana (Figure 5). This design was intentionally chosen to minimise setup costs, allowing the process to be managed in-house with limited technical support. Additionally, it enables internal updates to the LTP or other content on the application, reducing dependency on external resources and avoiding time delays. The submissions from the LTP have been used for advocacy and reporting purposes, such as contributing to the shadow report to the United Nations (UN) in 2023 (SADA 2023).

## Discussion

The results indicate that the PN and LTP provide a framework that can support people in using their voices to influence decisions that affect their lives, addressing barriers stemming from authoritarian power systems and ableism. Empowering marginalised groups involved creating inclusive, emotionally safe environments where individuals could reclaim their voices, fostering self-representation, agency and respect for diverse perspectives. At the same time, these processes were shaped by persistent challenges such as digital literacy gaps, unequal access to connectivity and the significant effort required to sustain participation.

In this context, collective social value may be generated when individuals are given platforms to share their experiences and knowledge. By recognising and validating their contributions, shifts in social participation and empowerment were observed, although these remain constrained by structural barriers. Within PN and LTP, parents’ voices were positioned as valuable assets, influencing policy, building social capital, fostering self-efficacy and contributing to meaningful outcomes, thereby building what has been termed a ‘voice economy’. Parent-led advocacy and empowerment models are identified as important drivers of disability inclusion, creating spaces for families to mobilise, build leadership and influence policy, while simultaneously facing the ongoing challenge of maintaining momentum in resource-limited contexts (Anderson & Bigby 2017; Burke & Hodapp 2016). By amplifying family voices and fostering mutual support, these models strengthen social capital and contribute to broader disability movements, though sustaining such efforts requires addressing structural inequities (Curran & Runswick-Cole 2014; Neely-Barnes et al. 2010).

Through various roles, such as network parents and parent champions, the strengths of network members were harnessed to implement the community care approach. Network parents played a catalytic role within the PN while also mediating challenges commonly faced in e-communities of practice, such as poor connectivity, lack of data and absence of smart devices (Kirik & Cetinkaya 2021; Zhu & Alamsyah 2022). While e-communities of practice expand opportunities for knowledge sharing and inclusive participation, they are equally marked by challenges of sustaining engagement and bridging digital divides (Dubé, Bourhis & Jacob 2006; Wenger, McDermott & Snyder 2002). Network parents attempted to address these barriers by visiting parents without smartphones or connectivity and assisting them in accessing the PN using their devices. They also filtered information between the provinces and SSE, guided parents in engaging in local languages within their provincial groups, and focused discussions on what was appropriate for a particular province (Gujar 2021).

Digital technologies provided opportunities to amplify marginalised voices and facilitate participation (Kirik & Cetinkaya 2021; Zhu & Alamsyah 2022). The PN and LTP used digital tools to help address barriers to empowerment through capacity building and access to information. However, limitations related to connectivity, affordability and digital literacy continued to restrict access for some families. The PN and LTP partially addressed this barrier through their operational framework, though uneven digital infrastructure remained a challenge. Similarly, the LTP’s capacity to store information allowed parents to access resources from the safety of their homes at their convenience, but ongoing support and training were often required. Consequently, while capacity building and support were expanded beyond time-bound activities, sustaining equitable participation required continuous attention (Ortiz et al. 2019; Baraka 2024).

The LTP’s offline functionality further mitigated some connectivity challenges, demonstrating potential in reducing barriers to voicelessness. Other studies have emphasised the importance of offline functionality in bridging the technological divide (Choung & Manamela 2018; Hillier 2018). Employing a technological solution (i.e., Andromo.com) that permits in-house design and allows for modifications as required by its users as their needs evolve within their environment has been valuable, though maintaining adaptability requires resources and technical support. The combination of innovation and technology available to not-for-profits (i.e., Kobo Toolbox) has made such adaptations feasible.

## Conclusion and recommendations

Drawing on the findings, the following were identified for future applications:

### Policy and governance

The PN and LTP frameworks supported social justice, enabling parents to influence critical aspects of their lives and fostering personal transformation. By providing accessible, virtual platforms for engagement, these tools enabled parents of CWD to reclaim their voices and actively participate in shaping policies. This approach not only amplified marginalised voices but also reinforced the importance of voice empowerment in achieving social justice.

#### Recommendations

- Policy developers and implementers should collaborate with social innovations like the PN and LTP to monitor the implementation of the WPRPD, ensuring stakeholder feedback is incorporated into the outlined datasets.
- The PN model should be explored as a framework for empowering other marginalised groups, with the LTP tailored to different contexts and policy focuses to expand inclusive decision-making.

### Platform design and accessibility

The PN and LTP tools created transformative spaces, empowering parents’ voices by providing an accessible and supportive environment. These tools allowed parents to reclaim their voices, gain confidence and express their opinions without fear of judgement. The PN and LTP tools also successfully utilised digital technology to create supportive spaces for expression, thereby reducing social exclusion and driving positive change.

#### Recommendations

- The LTP should be made more accessible to parents by simplifying how they identify the policy areas they want to monitor.
- Expand the roles of parents within provincial WhatsApp groups, using local languages to enhance accessibility and inclusivity.

### Research and future applications

Through voice empowerment, PWD and their families gained control, authority and confidence to make decisions and take actions that affect their lives. The PN and LTP fostered both personal and collective transformation, demonstrating the potential of digital innovation to amplify unheard voices and promote solidarity.

#### Recommendations

- Explore the concept of ‘voice economy’ as a means to intentionally include the voices of marginalised groups.
- Researchers and practitioners should investigate scaling the PN and LTP to other marginalised groups, focusing on building collaborative networks, enhancing capacity and influencing policy to reclaim unheard voices and promote social solidarity.
- Undertake explorative case studies that document parents’ experiences of using the PN and LTP, highlighting how they overcome challenges, apply new knowledge, develop skills and build support networks through participation.

### Limitations and challenges

While the PN and LTP demonstrated strong potential, several challenges emerged. Digital literacy barriers sometimes limited participation and engagement fatigue was noted among parents balancing multiple responsibilities. These constraints highlight the importance of ongoing support, capacity building and adaptation to sustain meaningful participation.

## Footnotes

1 Shonaquip Social Enterprise (SSE) is a South African social enterprise with a national footprint, delivering wheelchair services, assistive devices, and community-based training to promote mobility and participation for children with disabilities.

2 Cf. Shonaquip Social Enterprise (SSE 2021).
